# First detection of an ocellate octopus in the Revillagigedos ecoregion, a biodiversity hotspot located in the Tropical East Pacific Province

**DOI:** 10.3897/zookeys.986.53250

**Published:** 2020-11-05

**Authors:** Alejandra Valdez-Cibrián, Mariana Díaz-Santana-Iturrios, Víctor Landa-Jaime, Jesús Emilio Michel-Morfín

**Affiliations:** 1 Departamento de Estudios para el Desarrollo Sustentable de Zonas Costeras – CUCSUR, Universidad de Guadalajara. Melaque, Jalisco, Mexico. Gómez Farias 82, San Patricio-Melaque, Jalisco, C.P. 48980, Mexico; 2 Programa de Doctorado en Ciencias Biológico Agropecuarias, Universidad Autónoma de Nayarit, Xalisco, Nayarit, Mexico. Carretera Tepic-Compostela, Km 9, Xalisco, Nayarit, C.P. 63155, México; 3 Departamento de Ecología Aplicada– CUCBA, Universidad de Guadalajara. Camino Ing. Ramón Padilla Sánchez 2100, Predio las Agujas, Zapopan, Jalisco, 45510, Mexico

**Keywords:** Benthic octopus, Cephalopoda, Octopod, synonym, Tropical Pacific

## Abstract

The biodiversity of mollusks, particularly cephalopods, has not been exhaustively determined in the Revillagigedos ecoregion, which is a biodiversity hotspot for several marine groups located in the Tropical East Pacific Province. In our study, we detected and examined ocellate octopuses from Socorro and Clarion Islands, and determined their identity using morphological criteria and molecular data from two mitochondrial genes (COIII and COI). The taxon identified was *Octopus oculifer*, a species considered endemic to the Galapagos Archipelago. In addition, according to our analyses, *O. mimus*, *O. hubbsorum* and *O. oculifer* are very closely related and may represent a species complex comprised of three morphotypes. We found that the evolutionary relationships among octopuses are not determined by the presence of ocelli. This study is the first to report a clade represented by ocellate and non-ocellate species, in addition, the identity of cephalopods in the Revillagigedos was determined with analytical support.

## Introduction

Octopuses are soft-bodied cephalopods of the order Octopoda Leach, 1818, which comprises 13 families with around 300 pelagic or benthic species (Jereb et al. 2016). Benthic octopuses are either holobenthic, inhabiting the sea floor during the whole life cycle, or merobenthic, with a planktonic distribution during early stages (Villanueva and Norman 2008; Sauer et al. 2019). The family Octopodidae d´Orbigny 1940 includes 13 ocellate species catalogued in two genera, *Octopus* Cuvier, 1797 and *Amphioctopus* Fischer, 1882 (Jereb et al. 2016). Ocellate species (*Octopus cyanea* (Gray, 1849), *Amphioctopus exannulatus* (Norman, 1993), *A. fangsiao* (d’Orbigny, 18391841), *A. kagoshimensis* (Ortmann, 1888), *A. mototi* (Norman, 1993), *A. neglectus* (Nateewathana & Norman, 1999), *A. rex* (Nateewathana & Norman, 1999), *A. siamensis* (Nateewathana & Norman, 1999), *A. ovulum* (Sasaki, 1917), *O. bimaculatus* Verrill, 1883 and *O. bimaculoides* Pickford & McConnaughey, 1949) inhabit the Indian, Indo-Pacific and northwestern Pacific Oceans, except for *O. maya* Voss & Solis, 1966 and *O. oculifer* (Hoyle, 1904), which are considered endemic to the Yucatan Peninsula and the Galapagos Archipelago, respectively (Jereb et al. 2016).

Ocelli are considered an important diagnostic trait within octopodids, and are defined as false eye-spots in the form of round or ovoid conglomerations of chromatophores that may possess an outer concentric dark or light ring and an iridescent blue, purple, gold, or green inner ring (Packard and Hochberg 1977; Jereb et al. 2016). For octopodids, diagnostic features are highly valuable and needed, mainly due to the increased number of taxonomic confusions that derive from overlapped morphological characters among species (Norman and Hochberg 2005). Perhaps the most outstanding example of this problematic aspect is *O. vulgaris* Cuvier, 1797, which is considered a complex species that is being disentangled into different species (e.g., *Octopus insularis* Leite & Haimovici, 2008) and morphotypes by using morphological and molecular approaches (see Gleadall (2016), González-Gómez et al. (2018) and Amor et al. (2019)). Octopodids from the northeastern Pacific are no exception, for instance, Pliego-Cardenas et al. (2014) suggested that *O. mimus* Gould, 1852 and *O. hubbsorum* Berry, 1983 could be conspecific, and Díaz-Santana-Iturrios et al. (2019) confirmed that *O. californicus* Berry, 1911 and *O. alecto* Berry, 1953 should be reassigned into new genera.

Determining the biodiversity of octopodids is relevant given that several species constitute fishery resources (4.8 million tons extracted during 2005–2014) (Sauer et al. 2019) or present aquaculture potential (Baltazar et al. 2000; Iglesias et al. 2000; Chapela et al. 2006), thus, it is important to implement species-specific conservation and management measurements, especially in poorly known areas such as islands. Many insular systems are biodiversity hotspots (Hazen et al. 2013), often difficult to access, which hinders the characterization of biodiversity. The Revillagigedo Archipelago is an insular system declared Biosphere Reserve since 1994, and later, in 2016, the World Heritage Committee included it in the World Heritage List of the United Nations Educational, Scientific and Cultural Organization; more recently, in 2017, it was declared National Park (CONANP 2004; SEMARNAT 2017; UNESCO 2019). The Archipelago is located in the Tropical East Pacific Province and the Revillagigedos ecoregion, according to Spalding et al. (2007). Revillagigedos is considered a biodiversity hotspot, at least for sea turtles, sharks, whales and giant manta ray (Dutton et al. 2014; Muntaner 2016; SEMARNAT 2017). Moreover, in Mexican waters, Clarion represents one of the five islands with the greatest marine species richness, and Socorro is one of the nine islands with the highest number of strict endemism (Koleff et al. 2009). Determining the identity of taxa in biodiversity hotspots can be informative to understand and monitor global biodiversity patterns, especially in groups that respond markedly to the current climate change, as is the case for cephalopods (Renema et al. 2008; Robinson et al. 2009; Rodhouse et al. 2014). We visited Revillagigedos during 2018 with the purpose of characterizing the coastal malacofauna of the Archipelago and found ocellated octopuses; thus, in this study, the objective was to identify these octopodids collected in Clarion and Socorro Islands through morphological comparisons and partial COIII and COI gene sequence analyses.

## Methods

**Figure 1.**
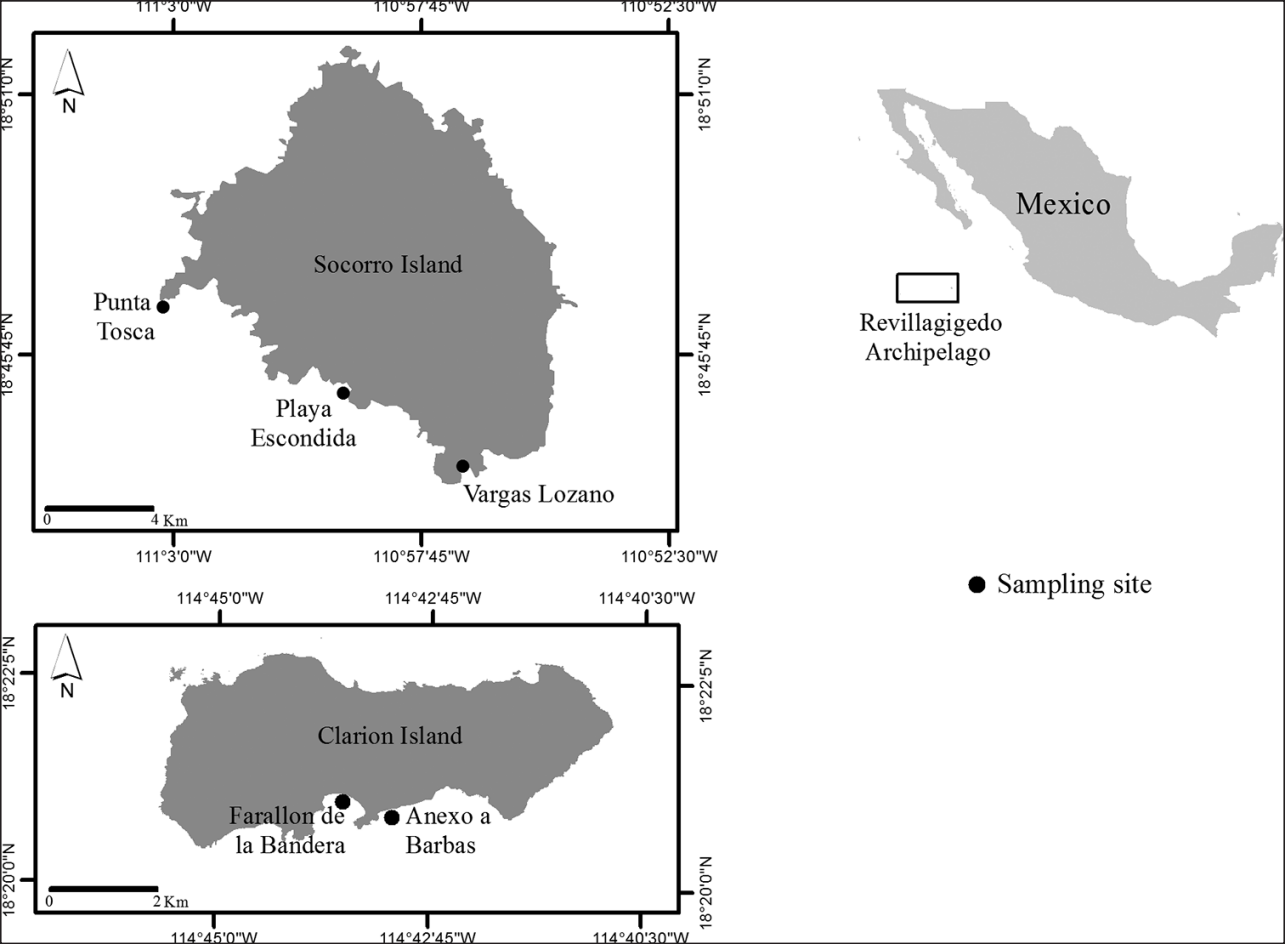
Study area. Sampling sites of octopuses from the Revillagigedo Archipelago.

The Revillagigedo Archipelago is located approximately 390 km southwest of the southern tip of the Baja California peninsula and 890 km west from Manzanillo harbor, between 17°39'19" and 20°00'31"N, and 110°04'41" and 115°28'17"W. The Archipelago is comprised of four volcanic islands: Roca Partida, San Benedicto, Clarion and Socorro (CONANP 2004; SEMARNAT 2017).The octopuses evaluated in this study were fished for self-consumption by crew of the Mexican Navy in the military bases of Clarion and Socorro in accordance with the regulations stated by SEMARNAT (2017) for the Revillagigedo National Park and the permit to develop scientific research on the malacofauna from the Archipelago (official document number: F00.1.DRPBCPN.DIR.PNR.-001/2018) during June, 2018. In this convenience sampling, a total of 49 individuals were collected in Socorro (*n* = 8) and Clarion (*n* = 41) islands (Table 1) (Fig. 1). Due to the sampling particularities (convenience sampling), we were not able to transfer whole specimens from the Archipelago to the laboratory, instead, the morphological identification and morphometric measurements were conducted *in situ* and a small piece of tissue of each specimen was preserved in vials with 96% ethanol. However, in order to account for a type specimen of our sampling, a whole specimen was frozen and transferred to the laboratory facilities, preserved in 96% ethanol and deposited as ICML-EMU-12678 in the Regional Collection of Marine Invertebrates (ICML-EMU), Instituto de Ciencias de Mar y Limnología, Unidad Mazatlán, UNAM, in Mazatlán, México.

**Table 1.** Data of octopuses from the Revillagigedo Archipelago.

Specimen number	DML (cm)	Sex	Maturity stage	Sampling site
1	6.5	M	I	Clarion Island
2	10	F	I	Clarion Island
3	7.5	F	I	Clarion Island
4	7	M	I	Clarion Island
5	8	M	I	Clarion Island
6	10	F	N/A	Clarion Island
7	8	F	N/A	Clarion Island
8	7.8	F	N/A	Clarion Island
9	7.5	F	N/A	Clarion Island
10	7	F	N/A	Clarion Island
11	8	F	N/A	Clarion Island
12	8	M	N/A	Clarion Island
13	10.5	M	III	Clarion Island
14	8.8	F	N/A	Clarion Island
15	7.3	F	N/A	Clarion Island
16	6.6	F	N/A	Clarion Island
17	3.1	F	N/A	Clarion Island
18	7.5	F	N/A	Clarion Island
19	6	F	N/A	Clarion Island
20	6.5	F	N/A	Clarion Island
21	10.5	M	III	Clarion Island
22	10	F	II	Clarion Island
23	7.5	M	III	Clarion Island
24	6.5	F	I	Clarion Island
25	6.5	F	I	Clarion Island
26	8	F	II	Clarion Island
27	9	F	II	Clarion Island
28	5.5	F	I	Clarion Island
29	7	F	I	Clarion Island
30	5.6	F	I	Clarion Island
31	5.7	F	I	Clarion Island
32	6.5	F	I	Clarion Island
33	6	F	I	Clarion Island
34	6.8	M	II	Clarion Island
35	6.8	F	II	Clarion Island
36	7.3	F	II	Clarion Island
37	8.3	F	II	Clarion Island
38	7.5	M	II	Clarion Island
39	6.9	M	II	Clarion Island
40	6.5	F	I	Clarion Island
41	6.5	F	I	Clarion Island
42	9	F	II	Socorro Island
43	6.3	F	II	Socorro Island
44	9	F	III	Socorro Island
45	8	F	II	Socorro Island
46	11.5	M	III	Socorro Island
47	11	M	III	Socorro Island
48	11	M	III	Socorro Island
49	12.8	F	III	Socorro Island

In Socorro Island, specimens were captured in each location by free and scuba diving using a hook; and in Clarion Island, octopuses were collected with a hook in the rocky intertidal during the lowest tide of each sampling site and day. Octopuses were sacrificed right after fishing. Individuals were sexed according to the presence (male) or absence (female) of a hectocotylized arm. Maturity stages for males and females were determined with the same criterion considered by Alejo-Plata et al. (2009). Octopuses were identified at the genus level using the morphological characteristics described in Jereb et al. (2016). For identification at the species level, the diagnoses of taxa of the genus *Octopus* that were reported for the Revillagigedo Islands by CONANP (2018) (*O. bimaculatus* and *O. hubbsorum*) and ocellate octopuses from the Eastern Pacific (*O. oculifer* and *O. bimaculoides*) were considered (Verrill 1883; Pickford and McConnaughey 1949; Berry 1953). The coloration patterns observed in the individuals were photographed with a Canon PowerShot D30 subaquatic camera. Images of morphological features of the octopus were recorded using a Canon EOS rebel T5 coupled to a stereoscopic microscope (Iroscope ES-24).

The dorsal mantle length (DML) and total weight (TW) were recorded to determine the length-weight relationship (LWR). Class intervals were determined following the Sturge’s rule. The following potential equation was employed to evaluate LWR:

*TW*= *a* * *DML^b^*

where: *TW* = dependent variable (total weight), *a* = coefficient of proportionality, *DML* = independent variable (dorsal mantle length), *b* = allometry coefficient (weight per unit of length).

The type of growth was estimated based on Student’s t-test for the “b” values obtained from the model and compared to a theoretical value of b=3 which represents isometric growth.

For the molecular approach, sequences deposited in GenBank (Table 2) of the octopus species reported for the Revillagigedo Islands, ocellate octopuses of the world and species of various octopod genera were compared to our sequences. Separate analyses of partial COIII and COI sequences were performed to support the morphological identification. For this research, four individuals (Specimens No. 44, 45 and 48 from Socorro Island and Specimen No. 38 from Clarion Island) were selected as representatives of the whole sample. DNA was extracted using the salt-extraction method. For COIII gene, a partial fragment was PCR amplified using the primers developed by Simon et al. (1994) as follows: amplifications were conducted in 12 μl reactions consisting of 2.4 μl of Colorless GoTaq Flexi Reaction Buffer (5× -Mg), 1.2 μl of MgCl2 (25 mM), 0.6 μl of dNTPmix (10 mM), 0.6 μl of each primer (10 μM), 0.1 μl of *Taq* polymerase (5 U/μl), 2.4 μl of combinatorial PCR enhancer solution (5×) (Ralser et al. 2006), 2.1 µl of Milli-Q H_2_O, and 2 μl of extracted DNA (32 ng/μl). The thermal cycler conditions were the following: 2 min at 94 °C for denaturation, followed by 35 cycles of 40 sec at 94 °C, 40 sec at 50 °C, and 1:30 min at 72 °C, and a final extension of 10 min at 72 °C. For COI gene, a partial fragment was PCR amplified using the primers developed by Folmer et al. (1994) as follows: amplifications were conducted in 15 μl reactions consisting of 2.1 μl of Buffer *Taq* (5× -Mg), 1.5 μl of MgCl2 (25 mM), 0.25 μl of each dNTP (10 mM), 0.9 μl of each primer (10 μM), 0.1 μl of *Taq* polymerase (5 U/μl), 3μl of combinatorial PCR enhancer solution (5×) (Ralser et al. 2006), 1.5 µl of Milli-Q H_2_O,and 4 μl of extracted DNA (32 ng/μl). The thermal cycler conditions were the following: 4 min at 94 °C for denaturation, followed by 30 cycles of 30 sec at 94 °C, 30 sec at 52 °C, and 1 min at 72 °C, and a final extension of 10 min at 72 °C. PCR products (COIII and COI) were analyzed with agarose-gel (1%) electrophoresis and stained with GelRed. All products amplified successfully, except for the COIII-gene sequence of Specimen No. 38, thus, it was not included in the respective analysis. Amplified products were sequenced in both directions with the same primers used for PCR (MACROGEN INC., South Korea).

All partial COIII and COI sequences were assembled and edited using BIOEDIT 7.2.6 software (Hall 1999). Edited sequences were deposited in the GenBank (Accession Numbers: MN259099–MN259105) (Table 2). All partial sequences of each gene were aligned using MUSCLE (Edgar 2004) in MEGA 7 software (Kumar et al. 2016). The phylogenetic relationships among octopodids were reconstructed using Bayesian inference in MR. BAYES v3 (Huelsenbeck and Ronquist 2001) with the GTR+G+I model (Tavaré 1986) (selected by BIC in MEGA 7 software). The analysis was conducted with four default heated chains, running 1 million MCMC iterations and saving at every 1000^th^ generation. The first 1000 trees were discarded as burn-in. Inter-specific genetic distances were estimated by Kimura-2-parameter model (Kimura 1980) in MEGA 7 software.

**Table 2.** Accession numbers of sequences (COIII and COI) obtained from GenBank of the octopodid species and specimens evaluated in this study.

Species	Accession number COIII	Accession number COI
*Octopus maya*	GU362546.1	MH293049.1
	–	KX611862.1
*Octopus cyanea*	AB573224.1	AB191280.1
	AJ628220.1	MK593394.1
*Octopus oculifer*	AJ628235.1	–
*Octopus hubbsorum*	KF225011.1	KY985096.1
	KF225010.1	KF225005.1
*Octopus bimaculoides*	KF225012.1	KY985076.1
	X83104.1	KF225006.1
*Octopus bimaculatus*	KT335840.1	KY985047.1
	NC_028547.1	KT335828.1
*Enteroctopus dofleini*	X83103.1	AB191272.1
	FJ603531.1	AB477017.1
*Octopus insularis*	KX219649.1	KY492362.1
	KX219648.1	KX611859.1
*Octopus vulgaris*	JQ085601.1	AB052253.1
	FN424384.1	KU525767.1
*Octopus tetricus*	AJ628240.1	MH289829.1
	JX680530.1	AF000056.1
*Octopus fitchi*	MK450541.1	MK450541.1
	KT335844.1	KT335832.1
*Octopus mimus*	KT335842.1	KT335830.1
	KT314263.1	GU355923.1
*Amphioctopus exannulatus*	AJ628223.1	–
*Amphioctopus fangsiao*	AB573188.1	HQ846155.1
	AB573186.1	AB430517.1
*Amphioctopus kagoshimensis*	AB573193.1	HQ846125.1
	AJ628226.1	HQ846123.1
*Amphioctopus mototi*	AJ628233.1	–
*Amphioctopus neglectus*	MH899749.1	MH899749.1
*Amphioctopus ovulum*	AB573198.1	HQ846159.1
	AB573197.1	AB430524.1
*Robsonella fontaniana*	KT314259.1	KF774313.1
	KC792301.1	–
*Hapalochlaena fasciata*	AJ628210.1	MF440346.1
	AB573212.1	JN790685.1
*Abdopus aculeatus*	AB573185.1	GQ900726.1
	AJ628213.1	LT604981.1
*Ameloctopus litoralis*	AJ628207.1	HM104255.1
*Eledone cirrhosa*	HM104251.1	KM517898.1
	–	MH293107.1
*Bathypolypus sponsalis*	FJ603530.1	KX078469.1
*Muusoctopus longibrachus*	KM459494.1	KM459478.1
	KM459486.1	KM459478.1
*Vampyroteuthis infernalis*	NC_009689.1	NC_009689.1
Specimen No.38	–	MN259102.1
Specimen No.44	MN259103.1	MN259099.1
Specimen No.45	MN259104.1	MN259100.1
Specimen No.48	MN259105.1	MN259101.1

## Results

**Figure 2.**
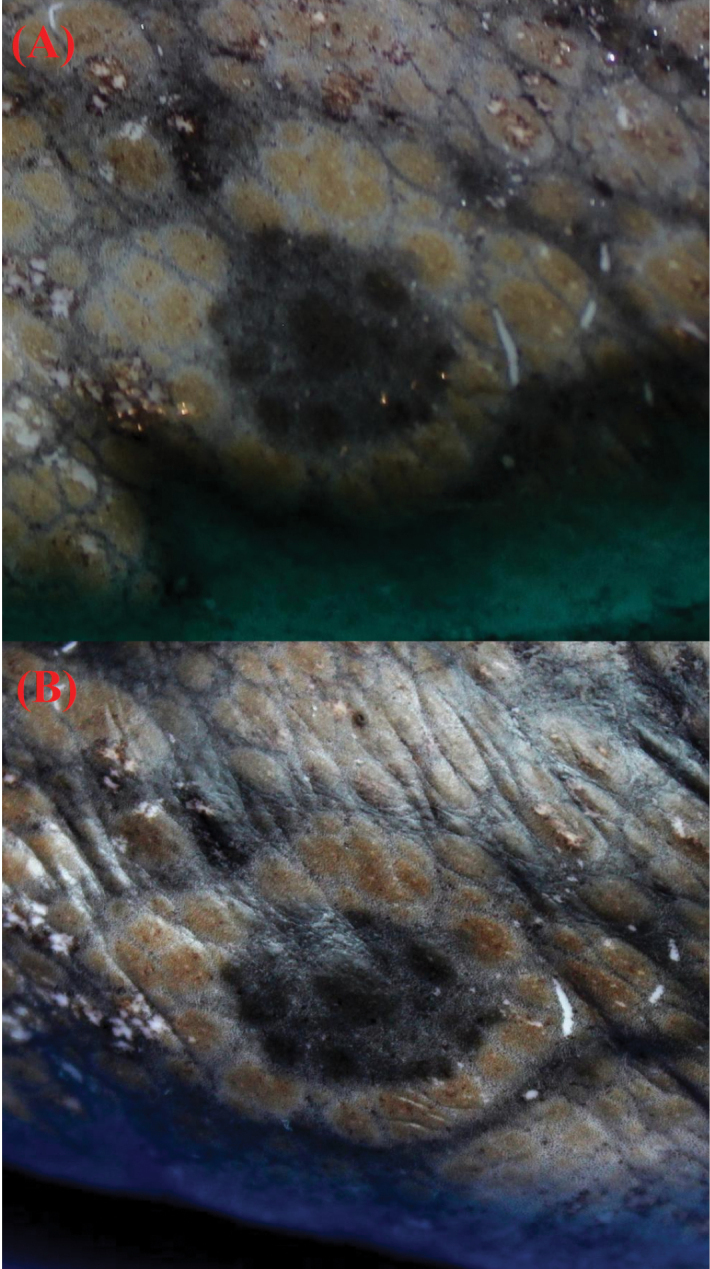
Ocellus of specimen No. 48 **A** defrosted **B** preserved in 96% ethanol.

The individuals analyzed belonged to the genus *Octopus* Cuvier, 1797; these presented an ink sac and suckers in a two-row arrangement. The specimens presented ocelli (Fig. 2) and were identified as *Octopus oculifer* according to the morphological characteristics specified in its original description (Table 3).

**Table 3.** Diagnostic features of species of the genus *Octopus* reported for the Revillagigedo islands and ocellate species of the world. Species identified in bold.

Species	Arm index	Arm formula	Sucker counts	Ocelli	Lamellae per demibranch	Funnel organ shape
*O. bimaculatus*	4 to 5	3>2>4>1	200 to 320	Yes	8 to 10	W
***O. oculifer***	**3.5 to 4.5**	**3>2>4>1**	**230 to 280**	**Yes**	**8 to 10**	**W**
*O. hubbsorum*	3 to 4	3>2>4>1	240	No	9 to 11	W
*O. bimaculoides*	3 to 3.5	3>2>4>1	140 to 190	Yes	8 to 10	W
*O. maya*	3 to 4.5	3>4=2>1	N/A	Yes	9 to 10	W
*O. cyanea*	4 to 6	4=3=2>1	450 to 500	Yes	9 to 11	W
*A. exannulatus*	2 to 3	3>4>2>1	120 to 190	Yes	8	W
*A. kagoshimensis*	2 to 3	4=3=2>1	150 to 170	Yes	8 to 9	W
*A. mototi*	2.5 to 3	3=4>2>1	140 to 170	Yes	9 to 11	W
*A. neglectus*	2 to 3	4=3>2>1	110 to 125	Yes	7 to 8	W
*A. rex*	2 to 3	4>3>2>1	134 to 184	Yes	8 to 9	W
*A. siamensis*	2 to 3	4=3>2>1	100 to 140	Yes	7 to 8	W
*A. ovulum*	N/A	4=3=2>1	59 to 70	Yes	15 to 17	W

### Diagnosis of the specimens collected in the Revillagigedo Archipelago

**Figure 3.**
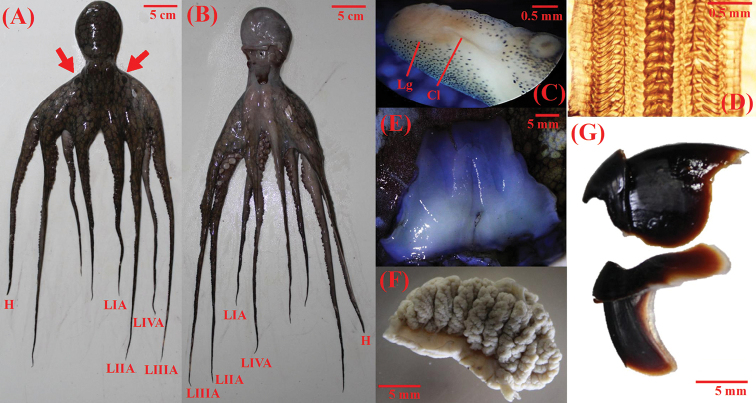
Morphological features. Morphological features of *Octopus oculifer* from the Revillagigedo Archipelago **A** dorsal view; H: hectocotylus, LI-IVA: left arms I-IV **B** ventral view; LI-IVA: left arms I-IV **C** ligula Lg and calamus Cl **D** H: hectocotylus, radulae **E** funnel organ shape **F** demibranch **G** upper and lower beaks.

**Figure 4.**
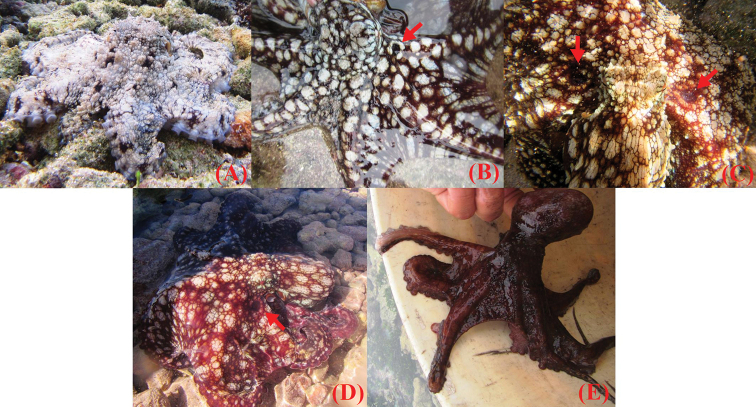
Coloration patterns. Coloration patterns observed in live individuals of *Octopus oculifer* from the Revillagigedo Archipelago **A** pale body with few reddish/brown spots randomly placed throughout the mantle and arms, entire body with a rugose aspect **B** brown and smooth body with large well-defined white ovals throughout mantle and arms **C** rugose and reddish body with large cream ovals of different size **D** red and smoother (still rugose) body with lesser number of cream ovals of different size **E** dark red body without ovals and a smooth skin.

**Figure 5.**
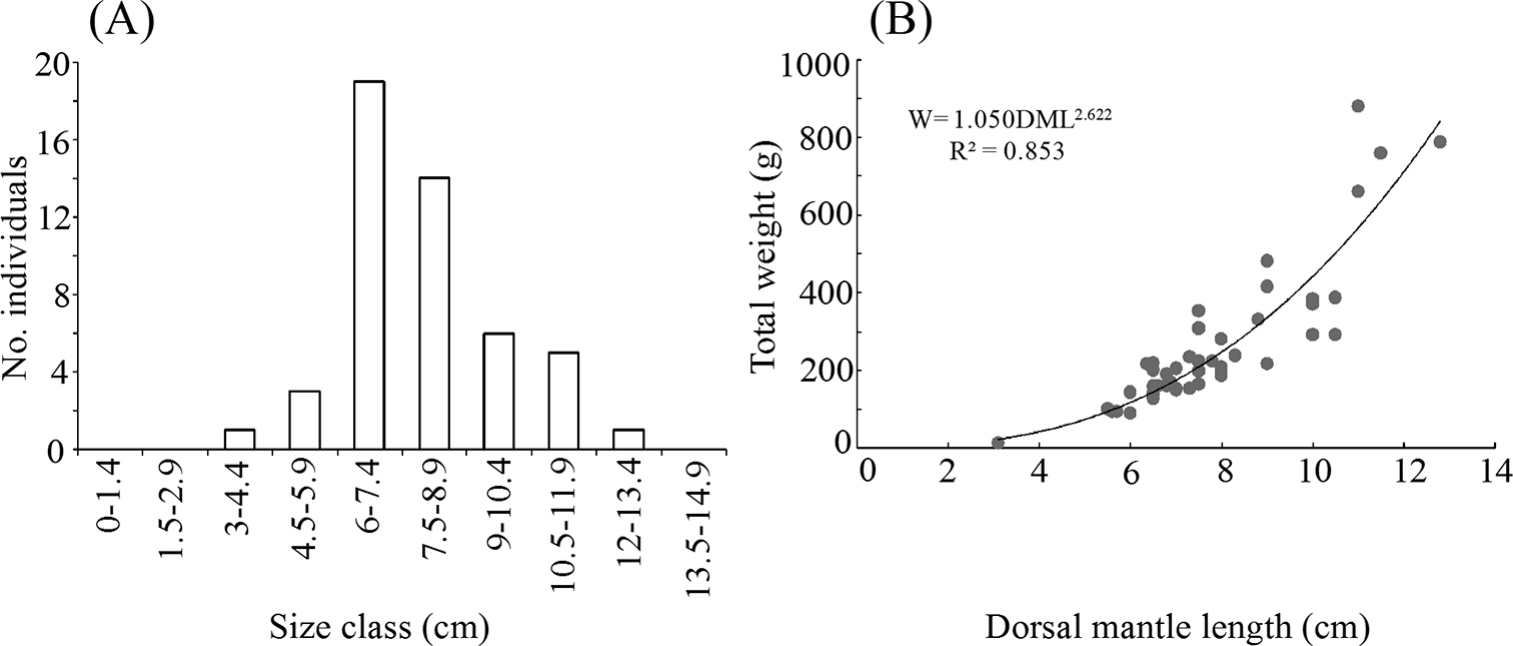
Length-weight relationship of *Octopus oculifer* from the Revillagigedo Archipelago **A** number of individuals per size class **B** length-weight plot.

The morphological features observed in the octopuses evaluated in this study are shown in Fig. 3A–G. Arm length ranged 3.5 to 4.4 times mantle length. Arm formula 3>4>2>1. Each arm with 230 to 280 suckers. Enlarged suckers on arms II and III. Gills with 10 lamellae per demibranch. Funnel organ W-shaped. Radula with 9 elements, 7 rows of teeth plus marginal plates. Right third arm of males hectocotylized (with 180 suckers). Ligula tiny, 0.3% of hectocotylized arm. Calamus small, 0.25% of hectocotylized arm. Upper beak: rostral tip blunt and thick; rostral curvature well-defined and extended anteroventrally; the hood extends in posterodorsal direction; crest curved dorsoposteriorly; dorsal portion of the lateral wall sharply angled towards the tip of crest; wing and shoulder compressed posteriorly into an almost vertical position; jaw angle and edge concave ventrally. Lower beak: pointed rostral tip; jaw edge extends in moderate slope posteroventrally; wing fold slightly angled; wing extended in dorsoposterior direction; the dorsal edge raised in the central portion; wall moderately curved posteroventrally towards the tip; free corner of the wall blunt; ventral edge of the wall curved. Color: five coloration patterns were observed in live individuals (Fig. 4), from pale and rugose body with few reddish-brown spots to completely dark-red smooth body without ovals. False-eye spots (ocelli) present as purplish black spot with a small pale central spot; ocelli are bound in an outer pale ring. The individuals analyzed in this study presented isometric growth (b = 2.62; t = 2.38; *p* = 0.07) (Fig. 5).

**Figure 6.**
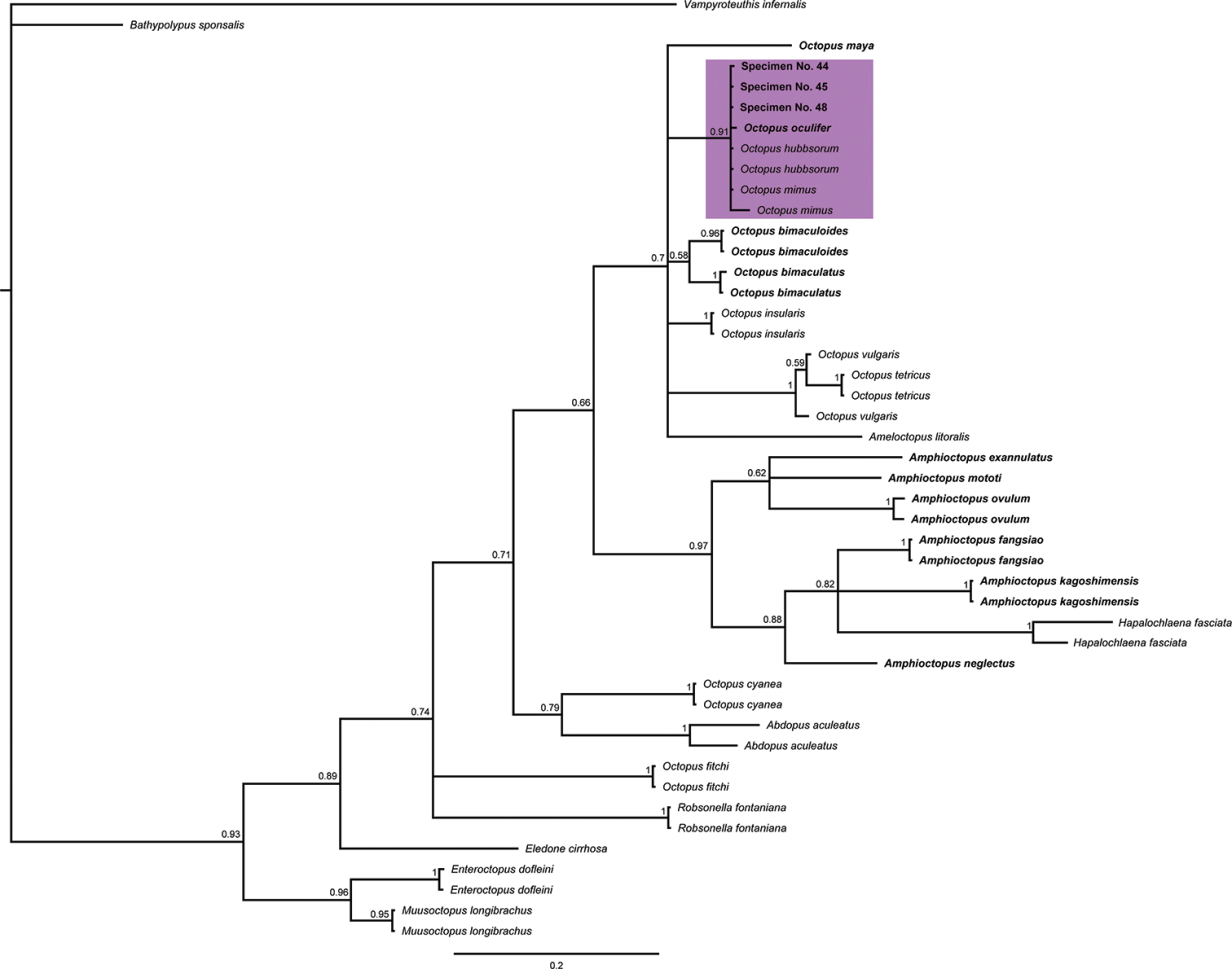
COIII Molecular phylogeny. Molecular phylogeny of COIII-gene sequences (273 bp: 136 variable and 137 conserved) of ocellate and non- ocellate octopus species. Ocellated octopuses are bold-faced. Purple rectangle indicates the clade containing the specimens evaluated in this study.

**Figure 7.**
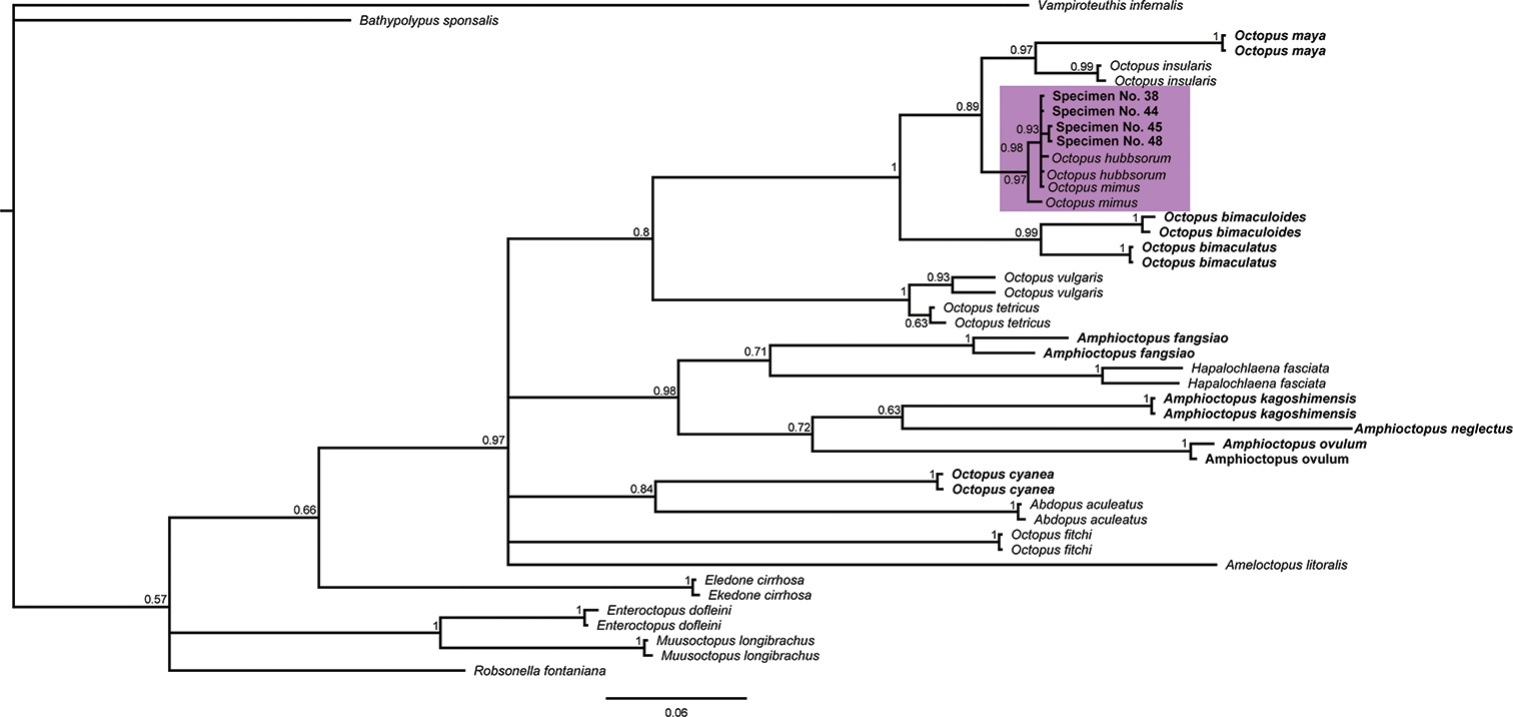
COI Molecular phylogeny. Molecular phylogeny of COI-gene sequences (474 bp: 193 variable and 281 conserved) of ocellate and non- ocellate octopus species. Ocellated octopuses are bold-faced. Purple rectangle indicates the clade containing the specimens evaluated in this study.

The phylogenetic trees of COIII and COI-gene sequences showed that the specimens from the Revillagigedo Archipelago belong to a clade associated with the ocellated octopus *O. oculifer* and the non-ocellate octopuses *O. hubbsorum* and *O. mimus* (Figs 6, 7). Similarly, the octopuses from the Revillagigedo Archipelago and sequences regarded as *O. oculifer*, *O. hubbsorum* and *O. mimus* presented low genetic distance (<1%) (Tables 4, 5). In addition, lower genetic distance was observed between ocellated and non-ocellated octopuses than between non-ocellated octopuses of the same genus (*e.g*., *O. insularis* and *O. bimaculatus* COIII=8.4% and COI=9.7% vs *O. insularis* and *O. vulgaris* COIII=11.7% and COI=11.8%) (Tables 3, 4). Closer relationships were also observed between ocellated and non-ocellated octopuses than between non-ocellated octopuses in the phylogenetic trees (e.g., clade comprising the individuals collected in the Revillagigedo Archipelago and clade containing *Hapalochlaena fasciata*) (Figs 6, 7).

**Table 4.** Genetic distances among octopuses collected and reported in the Revillagigedo Archipelago (RA) and ocellate and non-ocellate octopuses of the world estimated for a fragment of COIII gene sequences. *Octopus maya* (*Oct may*), *Amphioctopus exalatus* (*Amp exa*), *Amphioctopus fangsiao* (*Amp fan*), *Amphioctopus kagoshimensis* (*Amp kag*), *Amphioctopus mototi* (*Amp mot*), *Amphioctopus neglectus* (*Amp neg*), *Amphioctopus ovulum* (*Amp ovu*), *Octopus cyanea* (*Oct cya*), Our specimens (*Our spe*), *Octopus oculifer* (*Oct ocu*), *Octopus hubbsorum* (*Oct hub*), *Octopus bimaculoides* (*Oct bdes*), *Octopus bimaculatus* (*Oct btus*), *Enteroctopus dofleini* (*Ent dof*), *Octopus insularis* (*Oct ins*), *Octopus vulgaris* (*Oct vul*), *Octopus tetricus* (*Oct tet*), *Octopus fitchi* (*Oct fit*), *Robsonella fontaniana* (*Rob fon*), *Hapalochlaena fasciata* (*Hap fas*), *Abdopus aculeatus* (*Abd acu*), *Ameloctopus litoralis* (*Ame lit*), *Eledone cirrhosa* (*Ele cirr*), *Bathypolypus sponsalis* (*Bat spo*), *Muusoctopus longibrachis* (*Muu lon*) and *Octopus mimus* (*Oct mim*).

Sp	*Oct*	*Amp*	*Amp*	*Amp*	*Amp*	*Amp*	*Amp*	*Oct*	*Our*	*Oct*	*Oct*	*Oct*	*Oct*	*Ent*	*Oct*	*Oct*	*Oct*	*Oct*	*Rob*	*Hap*	*Abd*	*Arne*	*Ele*	*Bat*	*Muu*	*Oct*
	*may*	*exa*	*fan*	*kag*	*mot*	*neg*	*ovu*	*cya*	*spe*	*ocu*	*hub*	*bdes*	*bws*	*dof*	*ins*	*vul*	*tet*	*fit*	*fon*	*fas*	*acu*	*lit*	*cir*	*spo*	*lon*	*mim*
*Oct may*	0%																									
*Amp exa*	17.9%																									
*Amp fan*	15.5%	14.1%																								
*Amp kag*	19.9%	15.2%	12.0%																							
*Amp mot*	17.9%	13.5%	12.9%	17.0%																						
*Amp neg*	16.4%	15.5%	11.4%	14.7%	14.1%																					
*Amp ovu*	18.5%	15.0%	15.4%	14.7%	14.4%	14.2%																				
*Oct cya*	18.2%	17.0%	15.8%	18.2%	18.5%	19.9%	17.6%																			
*Our spe*	13.3%	15.7%	15.4%	18.7%	16.2%	16.2%	15.1%	14.6%																		
*Oct ocu*	13.5%	16.1%	15.8%	18.5%	16.4%	15.8%	14.7%	15.0%	0.4%																	
*Oct hub*	13.2%	15.8%	15.5%	18.8%	16.1%	16.1%	15.0%	14.7%	0.1%	0.3%																
*Oct bdes*	10.3%	17.0%	15.2%	19.1%	15.5%	14.7%	15.1%	15.2%	8.9%	9.1%	8.8%															
*Oct bws*	10.7%	15.5%	15.8%	19.1%	16.7%	15.0%	14.7%	16.1%	8.5%	8.7%	8.4%	4.8%														
*Ent dof*	20.8%	20.2%	17.9%	17.6%	19.6%	17.0%	19.9%	19.2%	17.7%	17.3%	17.6%	16.9%	17.0%													
*Oct ins*	11.1%	15.2%	14.4%	18.5%	16.1%	15.0%	15.0%	16.4%	8.3%	8.5%	8.2%	9.7%	8.4%	18.8%												
*Oct vul*	14.1%	16.7%	15.2%	19.4%	16.7%	16.7%	17.4%	17.0%	12.1%	12.3%	12.0%	11.6%	11.4%	21.0%	11.7%											
*Oct tet*	15.0%	17.3%	15.5%	20.2%	18.5%	15.8%	18.8%	18.8%	13.3%	13.5%	13.2%	12.9%	12.2%	21.4%	12.3%	3.4%										
*Oct fit*	21.4%	18.5%	17.9%	17.9%	16.1%	18.2%	19.1%	19.9%	18.3%	18.5%	18.2%	18.2%	19.1%	20.5%	18.2%	20.1%	19.9%									
*Rob fon*	20.5%	19.4%	18.8%	19.4%	19.4%	21.4%	20.5%	18.8%	17.2%	17.6%	17.3%	18.5%	19.2%	19.8%	19.1%	19.1%	19.6%	20.2%								
*Hap fas*	19.5%	18.5%	13.2%	15.5%	15.7%	13.9%	15.4%	17.7%	17.5%	17.4%	17.4%	16.7%	16.7%	19.1%	16.7%	15.8%	16.3%	17.6%	20.5%							
*Abd acu*	18.9%	17.2%	17.0%	18.5%	18.9%	18.0%	17.7%	16.6%	15.3%	15.5%	15.2%	16.4%	17 6%	21.7%	16.0%	16.4%	17.7%	19.1%	19.9%	18.5%						
*Arne lit*	17.9%	17.9%	18.2%	19.6%	17.6%	17.0%	18.0%	17.9%	14.8%	15.0%	14.7%	13.5%	13.9%	18.6%	13.5%	14.2%	14.1%	19.4%	19.6%	18.9%	18.3%					
*Ele cir*	22.3%	22.3%	20.5%	22.0%	20.8%	19.9%	21.6%	19.6%	19.5%	19.6%	19.4%	21.4%	21.4%	21.1%	18.8%	20.7%	20.5%	22.0%	22.6%	18.5%	20.7%	22.9%				
*Bat spo*	19.4%	20.8%	16.1%	18.2%	20.8%	19.9%	18.6%	18.2%	18.1%	18.5%	18.2%	17.6%	19.5%	17.4%	18.5%	22.1%	22.6%	19.4%	18.5%	21.8%	22.0%	19.1%	20.5%			
*Muu lon*	21.1%	19.1%	16.4%	17.0%	17.6%	16.4%	18.5%	19.6%	18.1%	18.5%	18.2%	16.4%	17.3%	7.8%	19.6%	21.6%	22.3%	18.8%	19.4%	18.6%	21.4%	18.8%	19.1%	15.5%		
*Oct mim*	13.5%	15.4%	15.2%	18.8%	16.0%	16.1%	14.8%	14.5%	0.7%	1.0%	0.7%	9.1%	8.8%	18.0%	8.2%	12.2%	13.2%	18.2%	17.7%	17.7%	15.5%	15.1%	19.4%	18.2%	18.2%	0%

**Table 5.** Genetic distances among octopuses collected and reported in the Revillagigedo Archipelago (RA) and ocellate and non-ocellate octopuses of the world estimated for a fragment of COI gene sequences. *Octopus maya* (*Oct may*), *Amphioctopus exalatus* (*Amp exa*), *Amphioctopus fangsiao* (*Amp fan*), *Amphioctopus kagoshimensis* (*Amp kag*), *Amphioctopus mototi* (*Amp mot*), *Amphioctopus neglectus* (*Amp neg*), *Amphioctopus ovulum* (*Amp ovu*), *Octopus cyanea* (*Oct cya*), Our specimens (*Our spe*), *Octopus hubbsorum* (*Oct hub*), *Octopus bimaculoides* (*Oct bdes*), *Octopus bimaculatus* (*Oct btus*), *Enteroctopus dofleini* (*Ent dof*), *Octopus insularis* (*Oct ins*), *Octopus vulgaris* (*Oct vul*), *Octopus tetricus* (*Oct tet*), *Octopus fitchi* (*Oct fit*), *Robsonella fontaniana* (*Rob fon*), *Hapalochlaena fasciata* (*Hap fas*), *Abdopus aculeatus* (*Abd acu*), *Ameloctopus litoralis* (*Ame lit*), *Bathypolypus sponsalis* (*Bat spo*), *Eledone cirrhosa* (*Ele cirr*), *Muusoctopus longibrachis* (*Muu lon*) and *Octopus mimus* (*Oct mim*).

Species	*Oct*	*Amp*	*Amp*	*Amp*	*Amp*	*Oct*	*Our*	*Oct*	*Oct*	*Oct*	*Ent*	*Oct*	*Oct*	*Oct*	*Oct*	*Rob*	*Hap*	*Abd*	*Ame*	*Bat*	*E/e*	*Muu*	*Oct*
	*may*	*fan*	*kag*	*neg*	*ovu*	*cya*	*spe*	*hub*	*bdes*	*btus*	*dof*	*ins*	*vu*/	*tet*	*fit*	*Jon*	*fas*	*acu*	*lit*	*spa*	*cir*	*lon*	*mim*
*Oct may*	0%																						
*Amp fan*	15.0%																						
*Amp kag*	17.5%	13.9%																					
*Amp neg*	17.7%	15.8%	14.1%																				
*Amp ovu*	16.7%	13.6%	13.8%	14.5%																			
*Oct cya*	13.2%	15.8%	14.8%	16.2%	14.0%																		
*Our spe*	7.3%	14.0%	18.0%	17.2%	17.5%	14.8%																	
*Oct hub*	7.3%	14.2%	18.0%	17.4%	17.5%	14.6%	0.2%																
*Oct bdes*	10.9%	14.1%	15.5%	17.9%	16.9%	15.4%	9.6%	9.6%															
*Oct btus*	10.5%	14.0%	16.5%	18.1%	17.4%	14.2%	9.8%	9.8%	6.2%														
*Ent dof*	18.0%	18.9%	19.8%	17.5%	16.4%	16.2%	18.6%	18.6%	19.1%	19.1%													
*Oct ins*	7.7%	14.8%	16.5%	16.6%	16.1%	13.4%	5.9%	5.9%	9.7%	9.7%	17.6%												
*Oct vul*	14.3%	16.4%	16.0%	15.8%	17.6%	16.4%	12.8%	13.0%	14.3%	13.6%	18.6%	11.8%											
*Oct tet*	14.3%	15.7%	16.6%	15.1%	16.7%	15.1%	12.1%	12.3%	14.7%	14.5%	17.1%	11.8%	3.4%										
*Oct fit*	17.1%	15.6%	16.9%	17.3%	15.9%	15.9%	15.9%	15.9%	18.7%	18.8%	18.1%	16.7%	17.1%	15.8%									
*Rob Jon*	16.0%	17.5%	16.7%	15.6%	15.9%	16.6%	16.4%	16.1%	16.1%	17.3%	14.1%	15.8%	17.8%	16.8%	15.2%								
*Hap fas*	16.1%	14.1%	14.8%	17.1%	17.0%	16.1%	16.4%	16.4%	15.7%	16.0%	19.4%	15.5%	16.1%	15.3%	17.6%	17.4%							
*Abd acu*	16.8%	17.0%	17.3%	16.5%	18.2%	13.5%	16.5%	16.5%	18.2%	16.1%	20.9%	16.1%	16.9%	17.0%	16.4%	16.8%	16.8%						
*Ame lit*	18.6%	18.9%	19.0%	18.6%	16.8%	18.2%	18.9%	18.7%	18.0%	18.8%	17.3%	17.3%	17.7%	17.3%	16.2%	17.1%	18.2%	18.5%					
*Bat spo*	18.1%	17.3%	16.7%	16.0%	15.8%	15.8%	18.2%	18.2%	17.5%	18.4%	14.9%	17.5%	17.9%	16.8%	18.6%	14.1%	18.1%	17.3%	19.4%				
*Ele cir*	16.4%	16.9%	16.8%	19.7%	18.7%	15.1%	16.9%	16.9%	16.9%	17.2%	17.6%	17.0%	16.7%	14.9%	16.8%	15.3%	18.1%	16.0%	17.8%	16.4%			
*Muu lon*	19.3%	20.3%	18.6%	17.4%	16.9%	17.4%	20.5%	20.5%	21.0%	19.7%	9.4%	19.5%	18.5%	17.4%	20.0%	14.8%	19.1%	19.5%	19.8%	15.6%	17.4%		
*Oct mim*	7.4%	14.1%	17.9%	17.5%	17.4%	14.7%	0.5%	0.5%	9.5%	9.5%	18.7%	5.8%	12.9%	12.2%	15.8%	16.2%	16.2%	16.6%	18.8%	18.1%	17.0%	20.1%	0%

## Discussion

In this study we analyzed octopuses from the Revillagigedo Archipelago in an attempt to increase knowledge concerning cephalopods in this geographic area. We identified the octopuses to the species level primarily, according to their morphological attributes, and secondarily, using partial sequences of COIII and COI genes following Vecchione et al. (2017). The individuals were identified as *Octopus oculifer* (Hoyle, 1904) based on morphological and molecular examinations; however, the overlap of characters among the species reviewed in literature, especially between *O. oculifer* and *O. hubbsorum*, and the slight variation of arm formula in regard to original description (*i.e.*, 3>4>2>1 instead of 3>2>4>1), explained why Jereb et al. (2016) pointed out that these species are a confusing complex that needs to be carefully re-evaluated (Table 3). Unfortunately, there is no holotype designated for *O. oculifer*, however, the original description is well-delineated and it is the only official conduit that endorses the identity of the species. In our study, there was no reason to suspect that the octopuses evaluated belonged to a new (undescribed) ocellated species or to any ocellated octopus other than *O. oculifer*.

For octopodids, particularly for the species evaluated in this study, a great deal of the taxonomic confusion is related to the fact that the morphological attributes are not standardized among species and that the diagnoses of octopodids from the northeastern Pacific had not been updated since the species descriptions, except for the validation of *O. bimaculatus* and *O. bimaculoides* within the genus *Octopus* performed by Norman and Hochberg (2005), and the recent evaluation performed by Díaz-Santana-Iturrios et al. (2019) for eight species of the genus *Octopus*. Thus, in this study, we provided a detailed characterization of the specimens collected in the Revillagigedo Archipelago and described attributes that were not included in the diagnosis of *O. oculifer* such as images of anatomic parts and coloration patterns, description of beaks, and length-weight relationship. We suggest that such procedures should be addressed in further research concerning octopods from the Eastern Pacific in general.

The endemism and geographic distribution of *O. oculifer* restricted to the Galapagos Islands was well documented (Hoyle 1904; Edgar et al. 2004; Molina et al. 2004; Jereb et al. 2016); in fact, this area is the known distribution of the species to date. Contrastingly, in our research we found *O. oculifer* in the Revillagigedo Archipelago, Mexico, which is located approximately 3242 km northwest from the Galapagos Islands, Ecuador. Hence, with this information, in this study we report an increase of the distribution range of *O. oculifer*, which could be related to ocean-current patterns putatively used as dispersal mechanism during the paralarval stage, as was detected for larvae of the lobe coral *Porites lobate* Dana, 1846 in that same area (Galapagos and Revillagigedo Archipelago) (Reyes-Bonilla et al. 1999). Another explanation for this distribution expansion could be associated with changes in climatic conditions and similarities between original and new environments (Arkhipkin and Laptikhovski 2008; Stewart et al. 2014; Ramos et al. 2015), given that the Revillagigedos and the Galapagos Islands belong to the same realm (Tropical Eastern Pacific) according to Spalding et al. (2007). Thus, it is likely that the presence of *O. oculifer* in the Revillagigedo islands is related to the incipient climate change, which is beneficial for the abundance and distribution of cephalopods (Doubleday et al. 2016). It is worth noting, however, that the Revillagigedo Archipelago is a remote protected area, which limits human access, and therefore, the cephalopod diversity has been determined incidentally and with unreliable observations. As a result, octopods are only included as part of the mollusks and macroinvertebrate fauna of the Revillagigedo Archipelago in generalized taxonomic lists (i.e., González-Nakawaga and Sánchez-Nava 1986; Holguín-Quiñones et al. 1992; Ortega and Castellanos 1994; Ortega et al. 1995; Cabrera-Mancilla and Bautista-Moreno 2002; CONANP 2004; Friscione-Carrascosa 2005; Bedolla-Guzmán 2007; CONANP 2018) that do not specify the identification criteria or that employ taxonomic keys (*i.e.*, Keen 1971; Abbott and Dance 1998) that were not developed by specialized cephalopod taxonomists. These lists include identifications at the genus level (*Octopus* sp.), tentative determinations (Octopus cf. bimaculatus) and/or erroneous identifications, such as *Callistoctopus macropus* (Risso, 1826) (formerly *O. macropus*), from the Mediterranean and northwestern Africa (Jereb et al. 2016). Thus, it is more probable that the presence *O. oculifer* in the Revillagigedo Archipelago occurred much earlier in geological time due to different ocean current patterns putatively used as dispersal mechanism during the paralarval stage, as explained earlier, than during the current climate change, and that its presence was not noticed until this detailed revision.

In addition, the molecular analyses of partial COIII and COI sequences strongly evidenced that *O. oculifer*, *O. hubbsorum*, and *O. mimus* are very closely related (inter-specific distance lower than 1%) and it is highly likely that these taxa are conspecific and represent a species complex comprised by three morphotypes. However, our finding should be further confirmed with type material (when available) and complete re-descriptions must be performed to support that these taxa are the same species. Moreover, the closer evolutionary relationships found between ocellated and non-ocellated octopuses compared to the relationships among non-ocellated octopuses indicate that the presence of ocelli is not a determinant character in octopodid classification and therefore, it should not be considered a diagnostic attribute.

## Conclusions

In this research, we conclude that according to our integrative species identification, the specimens collected in the Revillagigedo Archipelago are *Octopus oculifer*. According to our molecular analyses the non-ocellate *O. hubbsorum* and *O. mimus* and the ocellate *O. oculifer* are very closely related and might constitute a single species comprised of three morphotypes. In addition, ocelli should not be considered a diagnostic attribute for octopodids but rather a supplemental character.
